# Safety vs. efficacy assessment of pharmaceuticals: Epistemological rationales and methods

**DOI:** 10.1016/j.pmedr.2014.08.002

**Published:** 2014-08-26

**Authors:** Barbara Osimani

**Affiliations:** University of Camerino, School of Pharmacology, P.zza dei Costanti, 62032 Camerino, Italy

**Keywords:** Safety assessment, Adverse drug reactions, Evidence-based medicine, Evidence hierarchies, Causal assessment, Hypothesis testing, Inductive methods, Bayesian epistemology

## Abstract

In their comparative analysis of Randomised Clinical Trials and observational studies, Papanikoloau et al. (2006) assert that “it may be unfair to invoke bias and confounding to discredit observational studies as a source of evidence on harms”. There are two kinds of answers to the question why this is so. One is based on metaphysical assumptions, such as the problem of causal sufficiency, modularity and other statistical assumptions. The other is epistemological and relates to foundational issues and how they determine the constraints we put on evidence. I will address here the latter dimension and present recent proposals to amend evidence hierarchies for the purpose of safety assessment of pharmaceuticals; I then relate these suggestions to a case study: the recent debate on the causal association between paracetamol and asthma. The upshot of this analysis is that different epistemologies impose different constraints on the methods we adopt to collect and evaluate evidence; thus they grant “lower level” evidence on distinct grounds and at different conditions. Appreciating this state of affairs illuminates the debate on the epistemic asymmetry concerning benefits and harms and sets the basis for a foundational, as opposed to heuristic, justification of safety assessment based on heterogeneous evidence.

## Highlights

• Benefit vs. harm: distinction of epistemic/practical constraints for safety assessment.
• Evidence standards in relation to distinctive epistemologies.
• Hypothesis testing vs. inductive methods of hypothesis confirmation.
• Probabilistic hypothesis of causality in safety assessment.
• Epistemological rationales for the acceptance of “lower level” and heterogeneous evidence.

## Introduction

Evidence standards are supposed to provide “quick and dirty rules” for assessing the quality of evidence, as a function of the greatest possible reduction of confounding and bias. Hence, randomized controlled studies are followed by comparative studies which are not randomized (e.g. cohort or case–control studies), and these are followed by reasoning about pathophysiologic mechanisms underlying the observed outcome. Expert judgment is regarded as the weakest form of evidence and put at the bottom of the hierarchy (see Howick, 2011, for a philosophical overview).

At present, no distinction is explicitly made concerning the role of such standards for assessing safety instead of efficacy. However, recent contributions by philosophers and health scientists have acknowledged the role of so called “lower level” evidence as a valid source of information contributory to assessing the risk profile of medications. Some of them are based on empirical surveys attesting that both randomized clinical studies and observational ones deliver the same incidence estimates for a series of risks associated with medical interventions, both pharmaceutical and surgical (Benson and Hartz, 2000, Golder et al., 2011, Papanikolaou et al., 2006). Others draw on various methodological considerations (Aronson and Hauben, 2006, Howick et al., 2009, Vandenbroucke, 2008). These suggestions have noteworthy implications when considering current emphasis on evidence hierarchies, since they imply an asymmetry in the way evidence of benefits and risks of health technologies should be evaluated. However such suggestions fail to be grounded on a sound epistemic basis and seem rather ad hoc, although intuitively correct. Thus my aim here is to present their epistemological underpinnings by relating them to the common partition into (statistical) hypothetico-deductive, abductive, and inductive(-Bayesian) approaches to scientific inference. Furthermore, I will point out a series of pragmatic constraints for which inductive rather than deductive approaches to scientific inference should be considered as better suited to the purpose of risk assessment.

## The rationales underpinning evidence hierarchies and alternative approaches: necessary vs. uncertain inference

Scientific inference may be categorized in two main typologies: on one side, inferences whose conclusion follows necessarily from the set of premises and “rules” involved (e.g. laws and initial conditions). On the other side, cases falling under the heading of “uncertain inference”, i.e. situations in which the conclusion is highly probable or plausible, but fallacious from a strictly logical point of view. The former generally fall into the category of deductive inference, whereas the latter are generally labeled with the umbrella category of “induction”. An additional form of uncertain inference falls under the heading of inverse induction (or abduction, as proposed by C.S. Peirce).^1^Both induction and abduction are rationally compelling but logically unwarranted methods of hypothesis confirmation. The distinction between them mainly consists in the former relying on probabilistic evidence, whereas the latter focuses on explanatory considerations. Current theories of scientific justification have coined an alternative term for abduction; this is “Inference to the Best Explanation” (also known as IBE, see Lipton, 2004); although not completely overlapping, the two concepts heavily rely on theoretical virtues such as simplicity/parsimoniousness (Ockham's razor) and informativeness/explanatory power as (imperfect indicators) of reliability: explanatory power of a theory is a mark of its truth. Deductive and inductive/abductive rationales underpin diverse methods of evidence evaluation for causal assessment as illustrated below.

### Hypothesis testing as a deductive approach to scientific inference

In classical hypothesis-testing, the result is expressed as the probability of observing the experimental result or more “extreme” results in the sample space (p-value), if the treatment makes no difference (so called null Hypothesis: H_0_). The underlying epistemology is hypothetico-deductive (Popper, 1992): one assumes an entailment relationship between lack of efficacy and lack of difference between treated and untreated group (H_0_ ➔ E). If the outcome shows a statistically significant difference (¬E), then the hypothesis of lack of treatment can be rejected (¬H_0_), following classical modus tollens:

$$\begin{array}{l} H_{0}\to E\\ \frac{\neg E\;;}{\neg H_{0}\text{.}} \end{array}$$

In order to be able to draw a causal inference from the observed result, one must be confident that the difference between the two comparison groups is due to the contribution of the investigated factor, and only to it, otherwise ¬E might be due (also) to some alternative cause. Blinding, intervention and randomization are essential instruments in warranting this causal claim (see also Papineau, 1993, Worral, 2007, Osimani, 2013a, Osimani, 2013b, Osimani, 2013c) and evidence hierarchies are based on such warrants of internal validity. The EBM paradigm has been developed as a way to meet the desideratum that efficacy should be evaluated on the basis of the “best evidence” available, where “best” refers to quality criteria mainly informed by the requirement of internal validity.

The focus on internal validity is evident also in allowed deviations from evidence hierarchies in specific cases, i.e. where “lower level evidence” such as case reports and observational data are considered sufficient evidence for causal claims *to the extent that other conditions warrant for lack of bias and confounding*as alternative to randomization, blinding and intervention. Glasziou et al. (2007) for instance, consider cases where the relation between treatment and effect is so dramatic that bias and confounding can be safely excluded even if studies are based on just observational evidence: these are represented as phenomena of sudden and drastic changes in the clinical/epidemiological pattern and are formalized in terms of signal to noise ratio. Howick et al. (2009) relax the requirement of dramatic effect and reduce it to the desideratum that the effect size be greater than the combined effect of plausible confounders. Vandenbroucke (2008) considers that unintended effects, qua unintended, are not known in advance, and thus also not known by the drug prescriber, who cannot calculate on them and thereby possibly bias treatment allocation. It follows that observational studies concerning adverse reactions do not suffer from confounding in the same way as observational studies for intended effects do.

### Uncertain inference: probabilistic and explanatory approaches

Non-deductive methods abandon the goal of outright hypothesis acceptance or rejection and track uncertainty while updating the degree of confidence in a given hypothesis upon new evidence by also taking into account background knowledge. This allows them to be more flexible with regard to the kind of evidence which is allowed to inform hypothesis confirmation and the methods for amalgamating it.

Within this framework, the two somewhat contending paradigms are constituted by probabilistic approaches to hypothesis confirmation (e.g. Bayesian epistemology) and abductive reasoning (also fleshed out as “inference to the best explanation”, Lipton, 2004).

Bayesian epistemologies (Howson and Urbach, 2006) insist on hypothesis confirmation rather than testing, and allow statistics to measure the degree of confirmation provided by evidence E to a given set of hypotheses H = {h_1_, …, h_n_}, by relying both on the likelihood of the evidence in relation to each hypothesis P(E / h_i_), as well as on the probability measure associated to each hypothesis prior to collecting the evidence, P(h_i_), and by updating it through conditionalization (or other means, depending on the specific Bayesian approach adopted). This distinguishes them sharply from frequentist statistics where the p-value measures instead the probability of observing the evidence obtained in the experiment (or “more extreme results”) if the hypothesis under investigation is false.

In the Bayesian paradigm the main requirement is that all available evidence is used (Carnap, 1947, Carnap, 1950): this is because all non-deductive logics are non-monotonic. Non-monotonicity is a phenomenon which characterizes defeasible reasoning, i.e. contexts where the addition of further data to the initial premises may invalidate some previous conclusion (Kyburg and Teng, 2001): formally, you may for instance have that the probability of hypothesis H is greater than its negation given evidence E: P(H/E) > P(¬H/E); but by adding another datum to the previous body of evidence, the opposite inequality may hold: P(H/E,F) < P (¬H/E,F). This may be illustrated by a diagnosis of celiac disease (H) with evidence of immune reactions to certain kinds of food (E), and then weakening of this hypothesis after a laboratory test (F).

In the IBE paradigm, hypotheses are justified by their explanatory power: the greater the amount of data the hypothesis is able to explain, the greater its plausibility. Thus, explanatory power is considered to be truth-conducive (Lipton, 2004). This paradigm is seldom explicitly adopted in causal assessment for health technologies; however it often underlies systematic reviews and qualitative reports, where heterogeneous evidence is combined in a narrative fashion.

The first important advocate of alternative approaches to statistical hypothesis testing was Sir Austin Bradford Hill with his most cited President's Address (Hill, 1965) inaugurating the Section of Occupational Medicine of the Royal Society of Medicine; that is, a discipline mostly concerned with exposure to hazards. After presenting his nine guidelines for detecting and assessing causal relationships he claims: “None of my nine viewpoints can bring indisputable evidence for or against the cause-and-effect hypothesis and none can be required as a sine qua non. What they can do, with greater or less strength, is to help us make up our minds in the fundamental question — *is there any other way of explaining the set of facts before us, is there any other equally, or more, likely than cause and effect*?” (emphasis added). Thus, Hill both refers to explanatory power and hypothesis likelihood as reliable grounds to justify causal judgments.

In recent times other authors have endorsed similar proposals. Aronson and Hauben (2006) put forward that “In some cases other types of evidence may be more useful than a randomized controlled trial. Combining randomized trials with observational studies and case series can sometimes yield information that is not available from randomized trials alone”. This idea is also at the basis of the recent proposal by Howick et al. (2009) to integrate evidence hierarchies with Bradford Hill criteria for causal inference (see also Stegenga, 2011). Vandenbroucke (2008) proposes to invert hierarchies for the purpose of evaluating unintended outcomes on the basis of Bayesian as well as abductive considerations (see Osimani, 2013a, Osimani, 2013b, Osimani, 2013c). In all these cases the implicit epistemic framework is non-deductive and requires all available evidence (Bayesian epistemology) — or as much as possible of it (explanatory paradigm) — to be accommodated in the assessment. Table 1 illustrates the epistemological rationales for the adoption of lower level evidence following the deductive vs. non-deductive categorisation of scientific inference.

The next section presents a case study which illustrates the different epistemologies underlying, often implicitly, the distinctive stances in evaluating evidence for causal assessment. Such debates may be clarified by making the respective epistemic framework explicit. Furthermore, with the help of this case study, four points will be illustrated as to show why non-deductive approaches fare better in safety assessment.

## Case study: acetaminophen and asthma

The case of acetaminophen and asthma emerged with a study by Varner et al. (1998) who detected a precise correspondence between the increase of asthma incidence and increased acetaminophen use as a substitute for aspirin (which had been recognized to be associated with Reye's syndrome). The trend leveled off in the 1990s, i.e. at a time when acetaminophen had already become one of the most widespread analgesics. The suspicion raised by this study was further investigated by Newson et al. (2000) who conducted an ecological study showing a significant relationship between per-capita sales of acetaminophen and asthma morbidity across countries. Subsequent investigations explicitly aimed to examine the hypothesis of causal connection between paracetamol and asthma (Newson et al., 2000, Lesko et al., 2002, Barr et al., 2004: McKeever et al., 2005, Karimi et al., 2006, Beasley et al., 2008, Beasley et al., 2011, Shaheen et al., 2008, Lowe et al., 2010, Amberbir et al., 2011).

Possible biological pathways underlying the causal hypothesis have also been investigated. Eneli et al. (2005) summarize these findings and present five possible (non-exclusive) causal pathways accounting for the role of acetaminophen in asthma exacerbation. An additional immunologic pathway has been hypothesized by Nassini et al. (2010), namely the production of neurogenic airway inflammation caused by the transient receptor potential ankyrin-1 (TRPA1). Farquhar et al. (2010) have proposed another possible acetaminophen–asthma mediating mechanism based on its antipyretic effect.

The evidence gathered so far in support of the hypothesis of causal association between acetaminophen and asthma has generated two opposite stances. On one side, a series of authors show some reluctance in accepting such evidence as a sufficient basis for practice change and for establishing a causal relationship between acetaminophen and asthma, on grounds that it does not result from randomized clinical trials (Eneli et al., 2005, Allmers et al., 2009, Johnson and Ownby, 2011, Karimi et al., 2006, Wickens et al., 2011, Chang et al., 2011). Particularly, these authors express the concern that the acetaminophen–asthma relationship may be explained by reverse causation, or confounding by indication. Other authors are less imperative on the matter but equally require or recommend the performance of adequately powered placebo-controlled trials to establish causation (Holgate, 2011, Henderson and Shaheen, 2013). On the other side, Martinez-Gimeno and García-Marcos (2013) recommend against a too liberal use of acetaminophen in children, while waiting for regulatory agencies to do their part and reconsider the safety profile of acetaminophen on grounds that the acetaminophen–asthma association appears to be stronger and more robust than any other endogenous or exogenous candidate. Beasley et al. (2011) assert that “*when the study findings are considered together with other available data*, there is substantive evidence that acetaminophen use in childhood may be an important risk factor for the development and/or maintenance of asthma” (p. 1570, emphasis added). An even stronger commitment to the hypothesis of causal association is expressed by McBride (2011) who, considering all the evidence available claims that evidence of causal association can by now be regarded as strong enough to warrant a change in prescription practice.

McBride justifies his claim by appealing to the consistency of interdisciplinary evidence: 1) strength of the association displayed in comparative studies; 2) robustness of association across geography, culture and age; 3) dose–response relationship; 4) coincidence of time trends in acetaminophen use and asthma increase; 5) lack of other equally strong causal explanations; 6) relationship between asthma epidemic and per-capita sales of acetaminophen across countries; and 7) plausible mechanism. McBride explicitly warns against the use of acetaminophen in children with asthma or at risk for asthma and claims that if further evidence is required, then this is for documenting product safety rather than the contrary.

### Methodological dissent on causal assessment and safety issues

The dissent concerning the best course of action among scholars is ultimately caused by differing epistemological views which are left implicit. Those recommending the performance of placebo-controlled RCTs are in line with the rationales underlying evidence hierarchies. Thus they insist on the elimination of any suspicion of confounding, especially confounding by indication (Henderson and Shaheen, 2013, Chang et al., 2011). On the other side there are those who point to the joint support of different and independent sources of evidence as a valid basis for dropping any need for RCTs, thus implicitly relying on alternative ways to justify causal claims. Indeed, there are several reasons for preferring the latter to the former approach when evaluating evidence for safety assessment (McBride, 2011, Martinez-Gimeno and García-Marcos, 2013):

1) The threshold for practice change is determined by the risk–benefit balance: the higher the risk in comparison to the therapeutic effect, the weaker may be the hypothesis of the causal link in order to change the marketing status of the pharmaceutical product (Osimani, 2007, Osimani, 2013a, Osimani, 2013b, Osimani, 2013c). For instance, if an analgesic is found to be associated with carcinogenic risk, then, given its modest therapeutic importance in comparison to the suspected risk, the strength of the hypothesis of the causal association is not required to be high in order to take countermeasures (such as drug withdrawal) since it is counterbalanced by an unfavorable risk–benefit relationship; even a very low probability of causal association may suffice to retire the product from the market. Hypotheses of causal relationships need not be rejected or accepted: it is sufficient that they are strong enough with respect to the risk with which the treatment is suspected to be associated. In the case at hand, by claiming that if further evidence is required, then this is for documenting product safety rather than the contrary, McBride implies that such threshold has been trespassed. By shifting the burden of proof, McBride assumes that, given the expected harm and benefit, the probability of causal connection between acetaminophen and asthma is high enough as to shift the balance against its use.
2) Following Holland (1986), hypothesis testing should be seen as a method developed in order to assess *effects* of causes, rather than *causes* of effects, with the latter rather than the former being the focus of risk detection. Indeed when testing efficacy one is interested in ascertaining what kinds of effects a given treatment produces and in proving that no other causes is at play. Instead the main point of risk detection is to explain observed adverse events and to identify their causes in the first place. As Rudén and Hansson (2008) analogously point out, the focus of research in risk detection is on false negatives, rather than on false positives; which means that the problem is failure to see causation, rather than discerning spurious from authentic causation. Hence, standards for assessing causal claims concerning side-effects should not be parasitic on those developed for assessing treatment efficacy/effectiveness;
3) Concerning side-effects, most information comes gradually through “lower level” kinds of evidence, and there comes a point where the signal strongly suggests causation without demonstrating it. In such cases, one cannot pretend to know nothing about the side-effects of intervention until one doesn't have an RCT proving causality; yet this is exactly what the epistemology underpinning evidence hierarchies asks one to do.
4) RCTs deliver limited and purposely decontextualized information; they have been developed in order to test fertilizers on plant growth (Fisher, 1935). The causal structure here is much closer to physical causality (Thompson, 2011) — plots of lands do not react to fertilizers in the same way as human beings absorb and metabolize drugs (pharmacokinetics, pharmacodynamics) — and is not as rich in feedback loops, threshold effects, interactive causality as complex biological systems are, where such phenomena are much more frequent and entrenched. Hence another important reason for preferring a more flexible view on causality and justification of causal claims is also related to the ontological complexity of biological mechanisms upon which one should intervene.

In sum, the complexity of the causal structure in biological domains, the need for flexible decision tools which require the possibility to act upon uncertainty, and the focus on risk/benefit balance, strongly suggest a paradigm change in the evaluation of evidence for harm.

## Conclusion

Different epistemologies grant different methodological actions and grant “lower level” evidence on distinct grounds and at different conditions, thus it is worthy and also crucial to bear in mind the criteria underlying the evidence constraints one imposes on oneself. Appreciating this state of affairs illuminates the debate on the epistemic asymmetry concerning benefit and harm assessment and sets the basis for a foundational, as opposed to heuristic, justification of evidential support for pharmaceutical harm. Particularly, granted that knowledge about the drug risks comes from different sources and grows cumulatively over the course of time, probabilistic methods of causal assessment which rely on evidence amalgamation are better suited to the purpose. Instead, hypothesis testing is ill suited for risk management and pharmacosurveillance in that it has been developed in order to address the issue of false positives, rather than false negatives, which are the main concern in safety assessment. In sum, categorical approaches are too rigid for safety assessment on the basis of probabilistic hypotheses and of heterogeneous evidence which possibly fails to meet their strict desiderata.

## Conflict of interest statement

The authors declare that there are no conflicts of interests.

## Figures and Tables

**Table 1:** Rationales for justifying “lower level” evidence, as proposed in recent contributions, in relation to the corresponding epistemological paradigms.

Recent proposals for the adoption of “lower level” evidence in causal assessment	Related methodological assumptions	Underlying epistemology	
Howick (2011) and Howick et al. (2009): effect size greater than the combined effect of plausible confounders. Glasziou et al. (2007): dramatic relation between treatment and effect (sudden and drastic change in the clinical/epidemiological pattern). Vandenbroucke (2008): unintended effects cannot bias allocation in that they are not known in advance, hence non-randomized studies may be equally reliable.	Hypothesis testing: likelihood of evidence if H_0_ is true (p-value) Internal validity (homogeneous populations and conditions with regard to all possible relevant causal factors): “*best evidence*” (EBM)	Hypothetico-deductive (statistical mode)	Deductive approaches
Vandenbroucke (2008): probability of hypothesis given (“lower level”) evidence and prior (theoretical) knowledge.	Bayes theorem, conditionalization *Requirement of total evidence* and coherence: all available evidence must be considered for inference to be valid	Bayesian epistemology	Uncertain inference: probabilistic and explanatory approaches
Stegenga (2011) and Howick et al. (2009): integration of BradfordHill criteria with standard EBM criteria. Aronson and Hauben (2006): evidence amalgamation.	1. Several heterogeneous indicators of causality; 2. Causal hypothesis is confirmed by the amount of data it is able to accommodate. The more evidence the hypothesis is able to explain the more reliable it is: *explanatory power* is truth conducive.	Evidence amalgamation (possibly based on explanatory considerations)

## Footnotes

1 Peirce introduced the term “abduction” first in “Deduction, Induction and Hypothesis” (1934, Collected Papers 2.623), then in the Cambridge Conferences (1898) and in the 1903 Harvard Lectures (with different semantic nuances: see also Thagard, 1988, Section 4.2.1).
